# Keeping the End in Mind: Reviewing U.S. FDA Inspections of Submissions including Real-World Data

**DOI:** 10.1007/s43441-025-00791-1

**Published:** 2025-05-24

**Authors:** Cheryl Grandinetti, Donna R. Rivera, Lee Pai-Scherf, Anna Choe, Paul G. Kluetz, Stefanie Kraus, Gabriel K. Innes, Kassa Ayalew

**Affiliations:** 1 Division of Clinical Compliance Evaluation, Office of Scientific Investigations (OSI), Office of Compliance, Center for Drug Evaluation and Research (CDER), US Food and Drug Administration (FDA), Silver Spring, MD USA; 2 Office of Oncologic Diseases, Center for Drug Evaluation and Research and Oncology Center of Excellence, US Food and Drug Administration, Silver Spring, MD 20993 USA; 3 Division of Rare Diseases and Medical Genetics, Office of Rare Diseases, Pediatrics, Urology, and Reproductive Medicine, Office of New Drugs, Center for Drug Evaluation and Research (CDER), US Food and Drug Administration (FDA), Silver Spring, MD USA; 4 Office of Regulatory Policy, Center for Drug Evaluation and Research (CDER), US Food and Drug Administration (FDA), Silver Spring, MD USA; 5 Office of Medical Policy, Center for Drug Evaluation and Research (CDER), US Food and Drug Administration (FDA), Silver Spring, MD USA

**Keywords:** Real-world data (RWD), Real-world evidence (RWE), Good clinical practice (GCP), Regulatory inspections, Data quality and integrity, Drug approval submissions

## Abstract

The increasing use of real-world data (RWD) to generate real-world evidence (RWE) presents unique opportunities and challenges for drug development and regulatory decision-making, particularly in the area of good clinical practice inspections. FDA typically focuses their application review-based inspections on pivotal studies that generate evidence submitted to support new drug and biological product applications. This focus applies regardless of the data sources used in those studies. In this article, we discuss the fundamental role of good clinical practice inspections in verifying the quality, integrity, and reliability of RWD used in regulatory submissions. Through case examples, we highlight specific challenges related to accessing RWD source records, assessing data quality, and evaluating processes for data curation, transformation, and analysis. Our experience underscores the importance of early engagement with regulatory agencies as well as the implementation of robust quality management practices throughout the study lifecycle. As RWD continues to shape the regulatory landscape, these case examples provide insights in navigating the complexities associated with submissions utilizing RWE for drug approval.

## Introduction

Real-world data (RWD) have the potential to generate real-world evidence (RWE) for use in regulatory decision-making about the safety and effectiveness of drugs, including biological products [1]. The Food and Drug Administration (FDA) defines RWD as data relating to the patient’s health status and/or the delivery of health care routinely collected from a variety of sources. Examples of RWD include data derived from electronic health records, administrative claims, and product and disease registries, as well as data generated directly from patients (e.g., digital health technologies used in the course of clinical practice). RWE is the clinical evidence about the usage and potential benefits or risks of a medical product derived from analysis of RWD. RWD can be used to generate RWE in a variety of study designs from randomized, controlled clinical trials to non-interventional (observational) studies [2, 3].

The FDA has a longstanding history of reviewing RWD to evaluate the safety of previously approved drugs [2–7]. In recent years, submissions including the proposed use of RWE to support regulatory decision-making in the context of effectiveness have increased. In accordance with the 21st Century Cures Act [8], FDA formally created a program [2] to evaluate the potential use of RWE to support the approval of new indications for previously approved drugs or biological products or to support or satisfy post-approval study requirements. FDA created a RWE framework to implement this multifaceted program that included establishing and updating internal processes for the review of RWE, engaging with external parties, and publishing a series of guidance documents on the use of RWE in regulatory decision making (Table 1) [2]. The RWE program also includes funding and reviewing various demonstration projects to improve the usefulness of RWD, including improving study design and data analysis [3, 9].

**Table 1:**
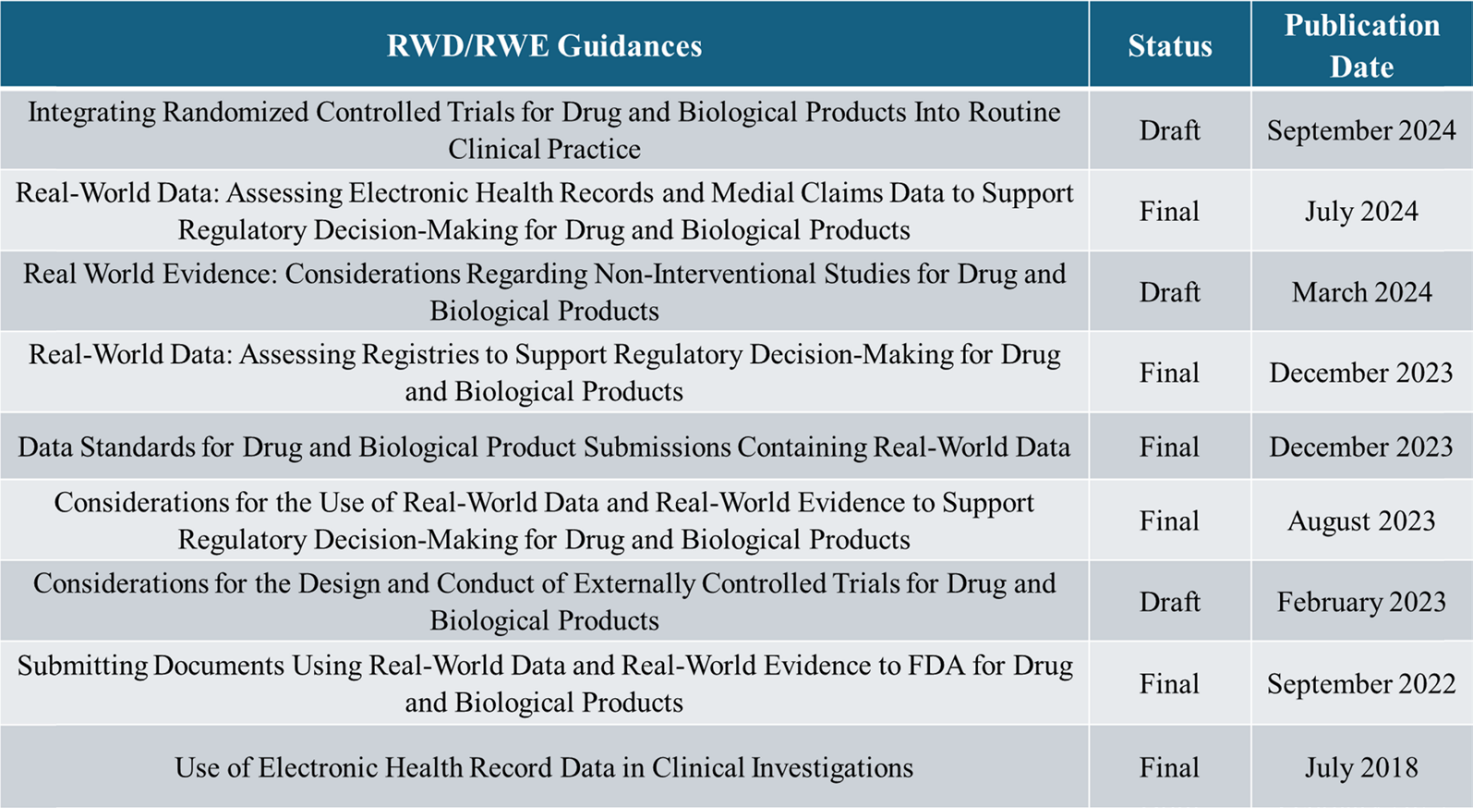
FDA Suite of RWD/RWE guidance documents

For links to RWD/RWE guidance documents and to ensure you have the most recent version, see https://www.fda.gov/science-research/science-and-research-special-topics/real-world-evidence

FDA requires substantial evidence of effectiveness based on adequate and well-controlled studies to support claims of effectiveness of a new drug or biological product, regardless of the data source or study design utilized [10]. For study data to be acceptable for regulatory decision-making, the data must be fit-for-use, that is, it must be relevant and reliable [11, 12]. RWD may be derived from a variety of sources and included in various study designs. Therefore, when planning studies using RWD, it is important to clearly define the study design and the data source.

Good clinical practice (GCP) inspections evaluate the quality of clinical studies and play a vital role in the FDA review and approval process for medical products [13, 14]. GCP inspections primarily focus on assessing study conduct and reporting to ensure the protection of participants and compliance with FDA regulations governing clinical trials. Additionally, a key aspect of these inspections is to assess the quality, integrity, and reliability of study data submitted to support a regulatory decision [15–17].

RWD are generally not collected in accordance with GCP standards, which complicates the assessment of data quality, integrity, and reliability for studies using RWD for several reasons [13, 18]. First, there is limited control over data collection methods used during clinical practice compared to methods used in traditional clinical trial settings that may lead to potential issues such as missing data, inaccuracies, and biases. Second, inconsistencies in how RWD are collected, recorded, and maintained across different systems, along with gaps in quality control monitoring, exacerbate these problems. Nonetheless, if RWD are to be used to inform regulatory decision-making, it is necessary to characterize data quality attributes, assess the fitness-for-use of the data to address the study question, and account for potential biases [19–21].

FDA’s decision to conduct an inspection that includes an assessment of RWD depends upon the purpose and contribution of the data to support the regulatory decision. For example, if the RWD are considered to provide pivotal evidence to the overall results of the study (e.g., if errors in RWD would substantively impact the regulatory decision), then an FDA inspection is more likely. Table 2 highlights the focus of FDA inspections for studies using RWD generating RWE [15, 16].

**Table 2:** Inspection focus of studies using RWD generating RWE

Essential study elements	RWD inspection focus
Protocol and protocol-related plans	• Assess study conduct to ensure protocol-related plans (e.g., statistical analysis plan, data management plan), and processes and procedures for data extraction, curation, transformation, and analysis were followed as pre-specified • Evaluate the measures implemented to minimize study bias and confounding and review study-related processes and procedures to ensure these measures were followed as pre-specified
Inclusion and exclusion criteria	• Review source records to ensure the pre-specified inclusion and exclusion were met for the study population
Cohort selection and attrition process	• Review documentation related to the attrition and selection process for the study analysis population (e.g., to assess for potential bias in the selection process)
Treatment regimen and dosing	• Review of treatment regimens and dosing is determined on a case-by-case basis. Since dosing may vary based on routine clinical practice, it may not be pre-specified in the protocol
Medication adherence	• Review of medication adherence is typically not a primary focus of the inspection, unless it is considered essential to support the regulatory decision. Medication adherence is generally not monitored in routine clinical practice during patient care
Data collection methods, including use of electronic systems and data collection tools (e.g., case report forms)	• Assess RWD for appropriate level of data quality and reliability (e.g., attributability, accuracy, consistency, completeness, and traceability) • Review and assess the data source (e.g., EHRs, registries) and data collection process within clinical practice to determine whether the quality of original data collected within clinical practice is fit for purpose for regulatory review • Review and assess the sponsor’s data collection tools (e.g., case report forms used to extract data from its RWD source) to determine their fitness-for-purpose for use in the study • Verify critical RWD submitted to support the regulatory decision against the source records to ensure that the data submitted to FDA match the source records
Prohibited and/or concomitant medications	• Review of prohibited and/or concomitant medications is determined on a case-by-case basis. Prohibited and/or concomitant medications may not be controlled or pre-specified in the protocol. In addition, prospective monitoring for prohibited and concomitant medications is generally not conducted within clinical practice
Protocol deviations	• Review documentation supporting data extraction, curation, transformation, and analysis processes and procedures to identify and assess any important process deviations that occurred that might have an impact on the overall study results • Review documentation for both reported and unreported protocol deviations and assess their potential impact on the overall study results
Protocol-specific training for individuals performing study-related activities	• Review documentation to determine that contract research organizations and other services providers received the appropriate training commensurate to their assigned study-related tasks and duties (e.g., data extraction, curation, transformation and analysis). Protocol-specific training does not apply to the health care providers who treated the patient within clinical practice
Data reconciliation during finalization of dataset	• Review the processes and procedures for data reconciliation, including documentation to support these processes and procedures to ensure they were followed as pre-specified • Assess the reconciliation process to verify that any changes to critical data are traceable to the source and that justifications for changes are documented, if applicable

Recent FDA inspections conducted in support of submissions using RWD in various study designs have identified unique challenges that can be categorized into three general areas related to: (1) access to RWD source records, (2) quality, integrity, and reliability of RWD, and (3) the RWD curation, transformation, and analysis processes. The objectives of this article are to describe inspection-related experiences and challenges, provide case examples, and provide insights regarding inspection readiness for submissions that plan to use RWD.

## Real-World Data Inspection Case Examples

### Case Study 1: Access to RWD Source Records

Access to source records is an essential part of the FDA inspection process, and in most cases, such access is necessary to verify the quality, integrity, and reliability of the RWD when the data provide evidence pivotal to the overall study results [15, 22]. Timely, on-site inspection of source records provides FDA with confidence that the data submitted are fit for use. Case example 1 illustrates challenges with sponsors providing FDA access to RWD source records during inspection, including issues related to informed consent and lack of sponsor agreements with third parties to permit FDA access to source records for inspection purposes.

In this case, an application submitted to FDA to support approval of an investigational product relied on efficacy data from an externally controlled trial. This trial featured a single arm interventional study with outcomes compared to an external control arm (ECA) derived from RWD. The ECA data were collected and curated by a third party from an international, disease-specific registry. Following the submission, the Sponsor informed FDA that due to General Data Protection Regulations and local privacy laws, providing FDA access to source records was not permitted without obtaining informed consent from each subject. In addition, due to internal policy of the third party and lack of an agreement between the sponsor and the third party, the sponsor informed FDA that an on-site inspection would not be permissible. The Sponsor subsequently proposed a targeted remote FDA audit of a subset of redacted patient source records maintained and retained by the third party, which the FDA subsequently deemed to be insufficient for data verification purposes. FDA emphasized the importance of having access to original source records or certified copies of these records from all patients included in the ECA as part of the FDA review process. The Sponsor and the third party subsequently established an agreement, and the Sponsor satisfied the requirements from the local ethics committee for FDA to have access to these records during inspection for data review and verification. De-identified records and selected certified copies of source records for all patients included in the ECA were made available for FDA review during the inspection of the third party.

### Case Studies 2 & 3: RWD Quality, Integrity, and Reliability

Issues with the quality, integrity, and reliability of RWD have been encountered during inspections, which have resulted in significant concerns about data reliability, ultimately limiting the utility of RWD in supporting regulatory decisions. While RWD may be susceptible to a myriad of errors and biases, the discussion herein is limited to errors and biases pertaining to the collection, extraction, and processing of RWD. It is important to not only understand the methods and tools that sponsors use to extract RWD from its original sources, but also to understand the original data collection methods and databases employed within clinical practice to gather the source data. For example, from an inspection perspective, the potential for the introduction of bias can occur because of data collection issues within clinical practice related to missing data and differences or inconsistencies in reporting endpoints or potential confounding variables. Thus, the FDA may assess these methods and databases during inspections to ensure data reliability.

Case example 2 illustrates how inconsistencies in data collection and assessment methods can impact the reliability of pivotal study data. To support approval of an investigational drug, the sponsor used a clinician-reported outcome as a primary efficacy endpoint in an externally controlled trial. This trial compared the results from a single-arm interventional trial to an ECA derived from an existing disease-specific registry. When reviewing the original source records, inspectors found significant inconsistencies in how the health care providers performed and scored the efficacy assessments in the ECA within clinical practice compared to how these assessments were performed and scored in the investigational arm by clinical investigators. These inconsistencies impacted the overall study results and resulted in data that were deemed not fit for use.

The third case example underscores the importance of evaluating relevant RWD sources early during the design of the study and during feasibility assessment. To support approval of an investigational drug, the Sponsor submitted an externally controlled trial that compared efficacy data from a single arm interventional study with an ECA derived from registry data. During FDA inspections, inspectors reviewed and verified the ECA data that had been curated and extracted by the sponsor from an existing disease-specific registry. The registry included information gathered in the course of clinical practice by healthcare providers who originally documented patient data in medical records. Healthcare providers transcribed the relevant information from the medical records onto excel spreadsheets, and these spreadsheets were sent periodically by email to the coordinating center for data entry into a registry. The sponsor subsequently curated and extracted data from the registry to electronic case report forms that were used to collect the extracted data. Audit trails were not available to track the original collection of data into the registry and no monitoring was performed (e.g., by the Sponsor) to determine if the data the Sponsor extracted from the registry accurately reflected the information documented in the original source records (i.e., medical records). Inspectors noted significant discrepancies (e.g., due to transcription errors and incomplete data) in the primary efficacy endpoint data between the source records and the patient-level data that were submitted to FDA to support the regulatory decision. This data collection and reporting process used to populate the registry limited the utility of the data to support the regulatory decision due to the significant discrepancies noted and the lack of traceability in the data collection process.

### Case Study 4: Data Curation, Transformation and Analysis Processes

Data curation, transformation, and analysis are key processes that can influence the quality and integrity of the RWD, and it is important to understand and verify these related processes and procedures. These processes and procedures typically would be pre-specified in the study protocol, statistical analysis plan, and data management plan. Submitting these plans for review and discussion with the FDA prior to initiating the study is beneficial [11, 12, 20–23]. Inspectors generally focus their assessments on the flow of data, starting with extraction from the original data source (e.g., electronic health record system) through to analysis and submission to FDA to ensure that “cherry picking” of data (i.e., selection of patients who would inflate benefit or reduce harm) did not occur; that pre-specified plans were followed; and that any reported deviations from these plans did not impact the study or introduce bias in the overall study results [12, 23].

The final case example serves to highlight challenges inspectors can encounter accessing and reviewing the documentation necessary to support the evaluation of processes and procedures for RWD curation, transformation, and analysis. In an application submitted to FDA to support approval of an investigational drug, the externally controlled trial included efficacy data from a single arm interventional study compared to an ECA derived from RWD. During inspection, records, including copies of medical records and related laboratory tests to support eligibility of the study population, were not available to evaluate how the ECA population was identified. The lack of available medical records challenges the ability to, among other things, assess selection bias, which could impact the approvability of the drug.

## Highlighting Challenges

Although FDA has provided considerations for the planning stages of the study using RWD [11], our inspectional experience highlights that certain aspects remain challenging. These key aspects include (1) the feasibility of providing FDA access to RWD source records (or certified copies), (2) addressing issues related to informed consent and record anonymization before the application is submitted to FDA, (3) evaluating RWD sources and databases used in the study and assessment of RWD quality, (4) implementing methods to ensure the quality of the RWD (e.g., conducting sponsor monitoring of the RWD after extraction from its source), and (5) maintaining and retaining documentation that enables the evaluation of the processes and procedures related to the RWD curation, transformation and analysis.

When RWD are deemed pivotal to evaluating study results or an evidentiary package, FDA may verify key RWD, including the evaluation of the source records (i.e., original, or certified copies). Access to original or certified copies of source records is key for inspection and data verification, ensuring that the patient-level data and information submitted from pivotal studies effectively support the study results that inform regulatory decisions [11]. FDA inspectors may need to access electronic systems (e.g., electronic health records, registries) that store source data and records to review and copy the relevant records [16, 20, 24]. If sponsors use third-party RWD providers, experience has shown that it is important to have agreements in place that allow FDA access to the necessary source records to verify the RWD [11]. It would also be important for sponsors to address informed consent and record anonymization issues before the application is submitted to FDA. Engaging early with Institutional Review Boards and Independent Ethics Committees to resolve these matters prior to submission is essential [11]. Failing to do so may lead to delays in inspections and approval processes or could impact the approvability of the drug if the RWD could not be verified.

To ensure that the RWD are of sufficient quality, it is important to identify and address potential sources of bias early in study design and evaluate potential concerns that might be associated with data collection during the feasibility assessment, where possible [11]. Because RWD can be generated from heterogeneous sources and not uniformly collected, data completeness can be a significant challenge. Data entry errors, inconsistencies, and discrepancies can significantly impact the accuracy of the data. Missing data may also lead to concerns about biased results, making it challenging to draw accurate conclusions. It would therefore be important to assess the RWD sources used to generate RWE during the feasibility assessment (which typically occurs early in the drug development program) to ensure data are of a sufficient level of quality and reliability.^11^ Of note, when assessing RWD sources, such as disease registry data submitted to support a regulatory decision, FDA inspectors typically: (1) verify the data collection methods and associated processes, (2) assess the training and processes in place for personnel to provide assurance that errors are minimized, and (3) determine whether the provenance of the data can be traced through audit trails and confirmed by source data [13, 15, 18, 20].

The RWD extraction method and data collection tools used by the sponsor should be fit-for-purpose and suitable for its use in the study (e.g., they should meet the specific protocol requirements for data collection) [13, 16]. Ideally, the original data collection method and databases used within clinical practice should incorporate the necessary measures to ensure data traceability (e.g., access controls and audit trails) [20, 21]. If the original data collection methods lack these fundamental controls, the potential risks of extracting data from such systems should be assessed (e.g., for the impact on data quality and integrity) to determine if the data are appropriate for regulatory purposes [24].

Additionally, it is important to document key decisions regarding selection of datasets, including the criteria for subject eligibility (e.g., prespecified eligibility criteria) [11, 13, 18, 23]. This documentation facilitates inspection and enhances transparency. It would be important to promptly identify any deviations from the pre-specified plans, protocols, and study procedures, and, when necessary, to evaluate and address them based on their significance [13, 18]. Risk-based quality management of the study is encouraged throughout the study lifecycle, beginning with the study’s design phase and continuing through RWD extraction from the source to analysis and reporting of results [13, 18]. Quality control activities, such as monitoring, should focus on maintaining quality and reliability of RWD throughout the study lifecycle [11, 13, 18]. Risk-based monitoring conducted by the sponsor is a helpful quality control tool to ensure that the RWD reported to FDA align with the source records and that the data are fit for regulatory review [11, 13, 18].

## Moving Forward

Use of RWD for regulatory decision-making depends on many factors, including the ability of the FDA to verify the quality and integrity of the data during an inspection. Experience during inspections highlights the benefit of engaging early with FDA and other relevant groups (e.g., third party RWD providers) when planning studies using RWD and RWE intended to support a marketing application [12]. These early discussions should cover critical study-related issues, including the scope and type of inspection needed. Proactively addressing potential challenges is paramount for timely inspection. Key considerations include ensuring compliance with FDA requirements for maintaining and retaining RWD source records that support the marketing application, as well as making these records available to FDA during inspection (as originals or certified copies). Additionally, it is important to address informed consent requirements, including anonymization of study records. Insights gained from these inspection case examples have identified shared learnings that can help parties while planning and designing studies incorporating RWD. Keeping the end in mind, if a study using RWD is designed to support FDA decision making, preparing for an FDA inspection is prudent.

## Data Availability

No datasets were generated or analysed during the current study.
